# A Novel Skeleton Compound from *Streptomyces canarius* SN0246: Isolation, Purification, and Structural Elucidation

**DOI:** 10.3390/microorganisms14071430

**Published:** 2026-06-30

**Authors:** Jiajun Chen, Chentong Yan, Pengfei Cui, Shijun Zhong, Zhiguo Yu

**Affiliations:** College of Plant Protection, Shenyang Agricultural University, Shenyang 110866, China; chenjiajun150@163.com (J.C.); yanchentong@163.com (C.Y.); 15730934739@163.com (P.C.); zsj1377064242@163.com (S.Z.)

**Keywords:** *Streptomyces canarius* SN0246, new skeleton compound, secondary metabolite, phytopathogenic fungi, antifungal activity

## Abstract

In this study, a compound with a novel chemical skeleton was isolated and purified from the fermentation broth of *Streptomyces canarius* SN0246. Its structure was elucidated using nuclear magnetic resonance (NMR) spectroscopy and high-resolution mass spectrometry (HRMS), and the compound was named canakingmycin. The in vitro antifungal activity of canakingmycin was evaluated against common crop pathogenic fungi, *Rhizoctonia solani*. The bioassay results showed that canakingmycin exhibited notable inhibitory effects on the mycelial growth of the phytopathogen, with the EC_50_ value of 76.13 μg/mL. This study enriches the structural diversity of secondary metabolites from Streptomyces and provides a promising lead molecule and scientific basis for the development of new bio-based fungicides.

## 1. Introduction

Plant fungal diseases represent major biological disasters causing global crop yield reduction and quality deterioration, posing severe threats to food security and sustainable agricultural development [1]. *Rhizoctonia solani* is a widespread plant pathogenic fungus in agricultural production [2,3,4]. They can cause a series of diseases including rice sheath blight, potato black scurf, and maize anthracnose, characterized by wide occurrence, rapid transmission, and heavy losses, resulting in enormous economic losses to agricultural production for a long time [5,6,7]. At present, chemical fungicides are still dominant in field disease control. However, long-term and excessive application has not only led to the increasingly serious development of pathogen resistance but also brought a series of problems such as pesticide residues and ecological environmental pollution [8]. Therefore, the development of efficient, low-toxicity, and environmentally friendly novel bio-based antimicrobial substances has become an urgent demand in the field of modern agricultural plant protection [9,10].

*Streptomyces* is an important producer of microbial-derived natural bioactive products [11]. Its metabolites possess remarkable advantages such as structural richness, novel skeletons, and diverse activities, serving as a core resource for discovering agricultural antimicrobial lead compounds [12]. Among them, secondary metabolites with novel skeletons are less likely to induce pathogen resistance due to their unique structures and novel action targets [13], which can break through the bottleneck of structural homogeneity of existing fungicides and provide key molecular templates for the research and development of new green pesticides, showing extremely high research and application value.

Microbial secondary metabolites exhibit significant characteristics including structural diversity, biological activity specificity, and producing strain specificity. Their chemical skeletons cover polyketides, nonribosomal peptides, terpenoids, alkaloids, and other types, and their complex chemical structures endow these products with abundant and unique biological activities [14,15,16,17]. In this study, *Streptomyces canarius* SN0246 was used as the research material. A polyketide compound with a new skeleton was isolated and identified by chromatographic separation combined with spectroscopic methods including nuclear magnetic resonance (NMR) and high-resolution mass spectrometry (HRMS), and it was named canakingmycin. The in vitro antifungal activity was evaluated by the mycelial growth rate method. The results showed that the compound exhibited significant inhibitory effects on *Rhizoctonia solani*, with the EC_50_ value of 76.13 μg/mL. This novel skeleton antimicrobial metabolite was discovered from *Streptomyces canarius* SN0246 for the first time. It not only enriches the structural diversity of secondary metabolites from *Streptomyces* but also provides an important lead molecule for the development of new bio-based agricultural fungicides. Meanwhile, it offers research ideas and scientific bases for further exploration of rare *Streptomyces* resources and their bioactive metabolites.

## 2. Materials and Methods

### 2.1. General Experimental Procedures

Nuclear magnetic resonance (NMR) spectra were recorded on a Bruker Avance-600 NMR spectrometer (Bruker BioSpin GmbH, Karlsruhe, Germany) at room temperature. High-resolution electrospray ionization mass spectrometry (HRESIMS) data were acquired using a Waters Xevo G2-XS Q-TOF mass spectrometer (Waters Corporation, Milford, MA, USA). Infrared (IR) spectra were obtained on a NICOLET IS50 spectrometer using the KBr pellet method with a scanning wavenumber range of 650–4000 cm^−1^ (Thermo Fisher Scientific, Waltham, MA, USA). Circular dichroism (CD) spectra were measured on a Jasco J-1500 spectropolarimeter with full-wavelength scanning (Jasco Corporation, Tokyo, Japan). Column chromatography was performed using 100–200 and 200–300 mesh silica gel (Qingdao Haiyang Chemical Co., Ltd., Qingdao, China). Sephadex LH-20 was purchased from GE Healthcare (Uppsala, Sweden). High-performance liquid chromatography (HPLC) analysis was carried out on an Agilent 1260 system (Agilent Technologies, Santa Clara, CA, USA) equipped with an Agilent ZORBAX Eclipse XDB-C18 column (250 × 4.6 mm, 5 μm). Semi-preparative HPLC was performed on the same Agilent 1260 system using a larger ZORBAX Eclipse XDB-C18 column (250 × 9.4 mm, 5 μm). All chemical reagents used in this study were purchased from Tianjin Fuyu Fine Chemical Co., Ltd. (Tianjin, China).

### 2.2. Actinomycete Material

*Streptomyces canarius* SN0246, isolated from soil of Liaodong Peninsula, China, was deposited in the China General Microbiological Culture Collection Center (CGMCC), Institute of Microbiology, Chinese Academy of Sciences. The deposit address is No. 3, Yard 1, Beichen West Road, Chaoyang District, Beijing, China. The accession number is CGMCC No. 20360, and the deposit date is 14 July 2020.

### 2.3. Tested Phytopathogenic Fungi

The pathogenic fungi used in this study were provided by the Laboratory of Microbial Secondary Metabolites, College of Plant Protection, Shenyang Agricultural University. All strains were cultured on potato dextrose agar (PDA) medium in an artificial incubator at 25 ± 2 °C with a relative humidity of 65–85%.

### 2.4. Fermentation and Extraction

*Streptomyces canarius* SN0246 stored at −80 °C was inoculated on Gauze’s No. 1 medium and activated for 5 days. The activated single colony was picked and inoculated into a 20 mL test tube containing 5 mL of sterilized ISP2 medium, followed by shaking incubation at 180 r/min and 28 °C for 48 h to prepare the first-stage seed culture. The first-stage seed culture was then transferred to a 250 mL Erlenmeyer flask containing 50 mL of ISP2 medium and incubated at 180 r/min and 28 °C for 12 h to obtain the second-stage seed culture. The second-stage seed culture was inoculated into 2000 mL Erlenmeyer flasks, each containing 400 mL of liquid Gauze’s No. 1 medium (composed of 20 g soluble starch, 1 g KNO_3_, 0.5 g K_2_HPO_4_, 0.5 g MgSO_4_.7H_2_O, 0.5 g NaCl, and 0.01 g FeSO_4_.7H_2_O per liter). Macroporous resin was added at 5% (*w*/*v*), and cultivation was carried out at 180 r/min and 28 °C for 7 days, with a total fermentation volume of 48 L.

The macroporous resin was collected by filtration, washed with distilled water, and dried at 28 °C for 48 h. The dried resin was eluted repeatedly by soaking in methanol, and the eluents were combined and concentrated to dryness. The concentrated extract was extracted multiple times with a solvent mixture of *V*_dichloromethane_:*V*_methanol_:*V*_water_ = 2:1:1. The organic phase was evaporated under reduced pressure to afford 8 g of crude extract.

The crude extract was subjected to silica gel column chromatography and gradient eluted with *V*_dichloromethane_:*V*_methanol_ = 100:0, 100:1, 100:4, 100:8, 100:16, 1:1, and 0:100. A total of 2000 mL of solvent was used for each gradient. Fractions were monitored by thin-layer chromatography (TLC) and combined according to similar profiles, finally yielding four fractions (A–D).

### 2.5. Isolation and Purification

Fraction B (1.3 g) was subjected to silica gel column chromatography and eluted with a gradient of *V*_dichloromethane_:*V*_methanol_ = 100:0, 99:1, 98:2, 97:3, 96:4, 95:5. The target fractions were combined based on TLC profiles to afford three subfractions: BA, BB, and BC.

Fraction BB was further purified by silica gel column chromatography using *V*_dichloromethane_:*V*_methanol_ = 98.75:1.25, 98.5:1.5, 98.25:1.75, 98:2 to obtain fraction BBA.

Subsequently, fraction BBA was chromatographed on a Sephadex LH-20 column eluted with *V*_petroleum ether_:*V*_dichloromethane_:*V*_methanol_ = 2:1:1. Similar fractions were combined according to TLC detection and further purified by semi-preparative HPLC under the following conditions: mobile phase *V*_methanol_:*V*_water_ = 35:65, column temperature 25 °C, flow rate 3 mL/min, detection wavelength 210 nm, and retention time (*t*R) = 70 min. Finally, 21 mg of canakingmycin was obtained.

### 2.6. Structural Characterization

Canakingmycin: yellow powder, odorless. The main characteristic absorption bands of IR spectrum measured by KBr pellet method are as follows: 3222 cm^−1^ (O-H stretching vibration), 2886 cm^−1^ (saturated C-H stretching vibration), 1684 cm^−1^ (carbonyl C=O stretching vibration), 1616 cm^−1^ (C=C double bond stretching vibration), 1165 cm^−1^ (C-O single bond stretching vibration), 1097 cm^−1^ (in-plane C-H bending vibration), 798–696 cm^−1^ (olefinic out-of-plane C-H bending vibration) (Figure S8). CD (CH_3_OH, 1 mm path length) Δε (nm): 243 (+37.23), 308 (−31.84), 337 (+56.61) (Figure S9). The ^1^H NMR and ^13^C NMR data are listed in Table 1. HR-ESI-MS: m/z 451.1748 [M + Na]^+^ (calcd for [C_24_H_28_NaO_7_]^+^, 451.1727) (Figure S7).

### 2.7. Antifungal Activity of Canakingmycin

The antifungal activity of the compound was determined via the mycelial growth rate method, with *Rhizoctonia solani* as the target pathogen. Canakingmycin was dissolved in a mixed solution containing 0.5% DMSO and 0.25% Tween-80, and then incorporated into molten PDA medium to prepare drug-containing plates (9 cm diameter) with serial final concentrations of 6.25, 12.5, 25, 50 and 100 μg/mL. The negative control group consisted of PDA medium supplemented only with sterile water, 0.5% DMSO and 0.25% Tween-80. Each treatment was set up with three biological replicates. Mycelial plugs of 7 mm diameter were cut from the edge of actively growing fungal colonies and placed in the center of each plate. The plates were incubated at 25 °C for 72 h. When the mycelial diameter of negative control plates reached approximately 8 cm, the colony diameters of all treatments were measured and recorded, and the half maximal effective concentration (EC_50_) value were calculated accordingly.

## 3. Results

### 3.1. Structure Elucidation of Compounds

High-resolution electrospray ionization mass spectrometry (HR-ESI-MS) yielded a sodiated molecular ion peak at m/z 451.1748 [M + Na]^+^ (calcd for C_24_H_28_NaO_7_^+^, 451.1727) ${\left[\alpha \right]}_{D}^{30}-100$. Combined with ^1^H NMR and ^13^C NMR data, the molecular formula of this compound was determined to be C_24_H_28_O_7_.

The ^1^H NMR spectrum of the compound showed signals for three hydroxy protons: *δ*_H_ 4.03 (s, 1H, 6-OH), 1.85 (s, 1H, 12-OH), and 1.70 (s, 1H, 13-OH) (Figure S1). Three olefinic methine protons were observed at *δ*_H_ 6.44 (dd, *J* = 10.1, 1.6 Hz, 1H, H-14), 5.62 (dd, *J* = 10.1, 5.3 Hz, 1H, H-15), and 6.57 (q, *J* = 7.3 Hz, 1H, H-20), which were assigned to the carbon signals at *δ*_C_ 131.3 (C-14), 123.4 (C-15), and 145.0 (C-20) by HSQC spectrum, respectively (Figure S4). Two olefinic methylene protons were detected at *δ*_H_ 5.42 (d, *J* = 3.0 Hz, 1H, Ha-5) and 5.15 (d, *J* = 3.0 Hz, 1H, Hb-5), corresponding to the carbon signal at *δ*_C_ 99.7 (C-5) in the HSQC spectrum. One oxygen-bearing methine proton was found at *δ*_H_ 2.90 (d, *J* = 9.5 Hz, 1H, H-12), which was correlated with the carbon signal at *δ*_C_ 80.9 (C-12). Three aliphatic methine protons were observed at *δ*_H_ 1.79 (m, 1H, H-8), 1.74 (m, 1H, H-11), and 2.44 (dd, *J* = 5.3, 1.6 Hz, 1H, H-16), assigned to *δ*_C_ 42.2 (C-8), 35.7 (C-11), and 50.7 (C-16), respectively. Four aliphatic methylene protons were present at *δ*_H_ 2.47 (m, 1H, Ha-9) & 1.78 (m, 1H, Hb-9), and 1.77 (m, 1H, Ha-10) & 1.16 (qd, J = 12.1, 4.0 Hz, 1H, Hb-10), corresponding to *δ*_C_ 21.9 (C-9) and 33.0 (C-10), respectively. Four methyl protons (corrected from “12”) were displayed at *δ*_H_ 2.05 (d, *J* = 7.3 Hz, 3H, H-21), 1.22 (s, 3H, H-22), 1.04 (d, *J* = 6.2 Hz, 3H, H-23), and 1.33 (s, 3H, H-24), which were attributed to *δ*_C_ 16.5 (C-21), 19.0 (C-22), 18.9 (C-23), and 22.0 (C-24) by HSQC spectrum. The full ^13^C NMR spectrum is shown in Figure S2.

The ^13^C NMR spectrum combined with the HSQC spectrum revealed the presence of: one ketone carbonyl carbon at *δ*_C_ 205.2 (C-18); one ester carbonyl carbon at *δ*_C_ 167.8 (C-1); four olefinic quaternary carbons at *δ*_C_ 127.6 (C-2), 144.9 (C-3), 152.2 (C-4), and 132.0 (C-19); and four sp^3^-hybridized oxygen-bearing quaternary carbons at *δ*_C_ 108.6 (C-6), 48.5 (C-7), 71.1 (C-13), and 85.0 (C-17).

**Figure 1:**
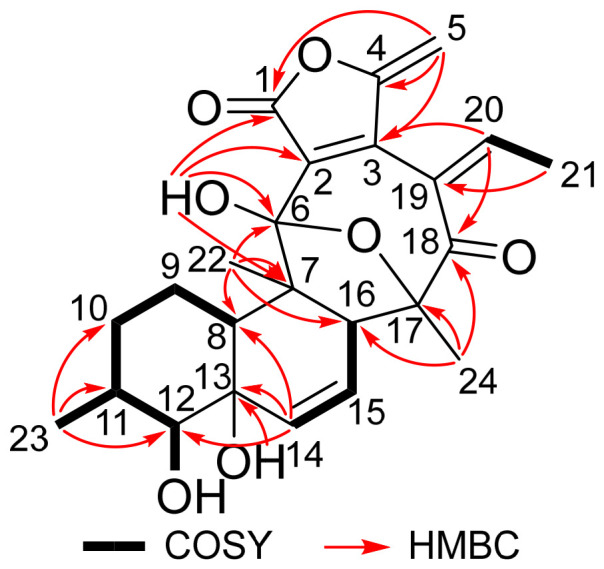
Key ^1^H-^1^H COSY and HMBC correlations of canakingmycin.

In the ^1^H-^1^H COSY spectrum (Figure S3), the following correlations were observed: H_a_-9 (2.47 ppm) showed correlation with H-8 and H_b_-10 (1.16 ppm); H-11 exhibited correlations with H_b_-10 (1.16 ppm), H-12, and H_3_-23; H-12 was correlated with 12-OH; H-15 showed correlations with both H-14 and H-16; H-20 was correlated with H_3_-21. Three spin-coupling systems were established as depicted by the bold solid lines in Figure 1.

**Figure 2:**
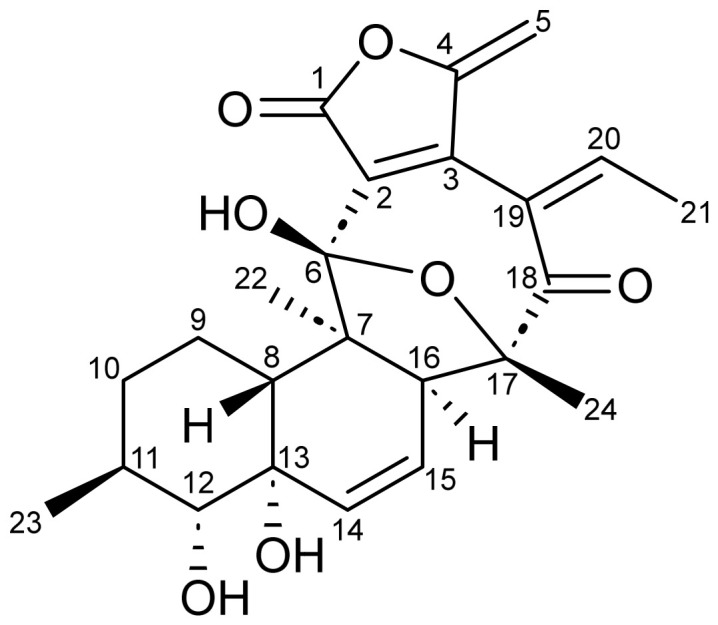
Planar structure of canakingmycin.

In the HMBC spectrum (Figure S5), key correlations were observed as follows: H_3_-23 showed correlations with C-10, C-11, and C-12; H-14 exhibited correlations with C-8, C-12, and C-13; H_3_-22 showed correlations with C-6, C-7, C-8, and C-16; 6-OH displayed correlations with C-1, C-2, C-6, and C-7; H_3_-24 exhibited correlations with C-16, C-17, and C-18; H-20 showed correlations with C-3, C-18, and C-19; H_3_-21 was correlated with C-19; H_2_-5 exhibited correlations with C-1, C-3, and C-4. Combined with the structural fragments deduced from the COSY correlations and the molecular formula C_24_H_28_O_7_, the planar structure of the compound was established and is illustrated in Figure 2.

**Figure 3:**
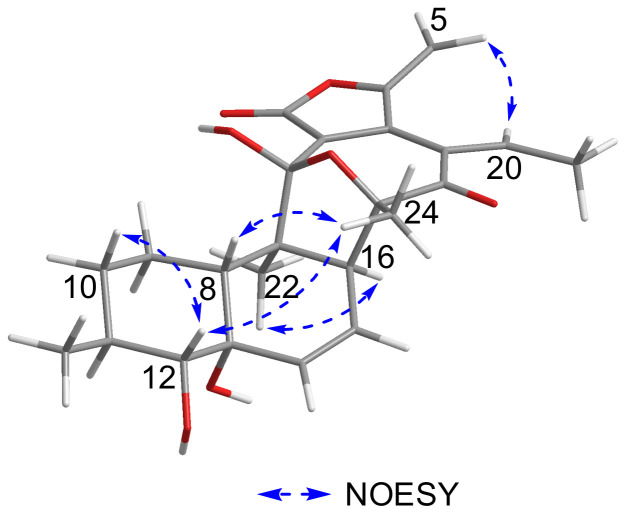
Key NOESY correlations of canakingmycin.

In the NOESY spectrum (Figure S6), H_3_-24 shows correlations with H-8 and H-12, while H_b_-10 (1.16 ppm) correlates with H-12, indicating that H-8, H_b_-10 (1.16 ppm), H-12, and the 24-methyl group are on the same side of the molecule (*β*-orientation). Furthermore, it is inferred that the 13-OH, 22-methyl, and H-16 also adopt the *β*-orientation; otherwise, H3-24 could not exhibit NOESY correlations with H-12. The observed NOESY correlation between H-22 and H-16 supports the above inference. Owing to the structural rigidity of the five-membered ring, the 6-OH is deduced to share the same orientation as the 24-methyl group, i.e., *β*-orientation. Based on the coupling constants ^3^*J*_10,11_ = 12.1 Hz between H_b_-10 (1.16 ppm) and H-11, and ^3^*J*_11,12_ = 9.5 Hz between H-11 and H-12, it is concluded that H-11 is in an axial bond (*β*-orientation) of the chair-conformed six-membered ring, and is trans to both H_b_-10 (1.16 ppm) and H-12. Thus, the relative configuration of this compound is proposed as shown in Figure 3.

### 3.2. Antifungal Activity Assay

**Figure 4:**
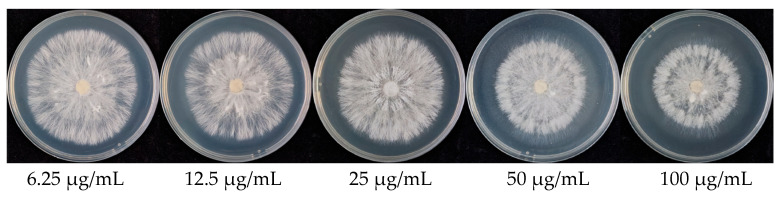
Inhibitory effects of canakingmycin on the mycelial growth of *Rhizoctonia solani*.

The results of the mycelial growth rate assay revealed that canakingmycin exhibited moderate inhibitory activity against *Rhizoctonia solani* under serial concentration treatments, with a calculated EC_50_ value of 76.13 μg/mL, as shown in Figure 4.

## 4. Discussion

This study isolated and identified a compound with an unprecedented skeleton from *Streptomyces canarius* SN0246. This is the first report of this metabolite from the species, which greatly enriches the structural diversity of secondary metabolites of *Streptomyces canarius*. The planar structure and relative configuration of the compound were unambiguously elucidated by HR-MS combined with one- and two-dimensional NMR spectroscopy, including HSQC, COSY, HMBC and NOESY. Meanwhile, IR and CD spectroscopy were also applied for auxiliary verification to further support the structural determination. With comprehensive structural evidence, the compound was named canakingmycin.

Possessing multiple oxygen-bearing quaternary carbons, hydroxyl groups and a distinctive ring system, canakingmycin implies a unique biosynthetic pathway and novel skeletal features, and thus holds great potential as a lead compound for pesticides or pharmaceuticals. In vitro antifungal bioassays indicated that canakingmycin possessed moderately antifungal activity against *Rhizoctonia solani*, with an EC_50_ value of 76.13 μg/mL.

*Rhizoctonia solani* causes severe diseases in rice, potato, maize and many other crops, leading to massive economic losses worldwide, causing widespread damage and substantial economic losses. The novel compound exhibited evident inhibitory activity against both pathogens, indicating its potential for development as an eco-friendly biopesticide. Its unique chemical skeleton is presumed to reduce the risk of inducing pathogen resistance, conferring advantages over conventional fungicides.

Although this work only conducted preliminary activity evaluation, it preliminarily demonstrates the great potential of rare actinomycetes for exploring novel bioactive metabolites. The discovery of this new-skeleton compound expands the chemical space of natural products derived from *Streptomyces* and provides a promising template for the development of new antifungal agents.

Further research is recommended, including the determination of absolute configuration, optimization of fermentation conditions, measurement of minimum inhibitory concentration (MIC) and exploration of antifungal mechanisms. These studies will lay a solid scientific foundation for the future development and application of canakingmycin.

## 5. Conclusions

In this study, a natural metabolite bearing an unprecedented skeleton was successfully isolated and structurally elucidated from the fermentation broth of *Streptomyces canarius* SN0246 using high-resolution mass spectrometry and nuclear magnetic resonance spectroscopy, and it was designated as canakingmycin. In vitro antifungal experiments demonstrated that canakingmycin exhibited moderate inhibitory activity against *Rhizoctonia solani*, with an EC_50_ value of 76.13 μg/mL. This finding enriches the structural diversity of secondary metabolites from *S. canarius* and provides a novel skeleton lead for the research and development of green agricultural fungicides. Moreover, it validates the significant potential of rare actinomycetes in exploring innovative bioactive natural products, thereby laying a fundamental basis for subsequent structural optimization and antifungal mechanism investigation of this compound.

## Figures and Tables

**Table 1:** ^1^H and ^13^C NMR spectral data for canakingmycin in CDCl_3_.

No	^1^H NMR	^13^C NMR
1		167.8
2		127.6
3		144.9
4		152.2
5	5.42 (d, *J* = 3.0 Hz, 1H)	99.7
	5.15 (d, *J* = 3.0 Hz, 1H)	
6		108.6
7		48.5
8	1.76–1.82 (m, 1H)	42.2
9	2.44–2.50 (m, 1H)	21.9
	1.75–1.81 (m, 1H)	
10	1.74–1.80 (m, 1H)	33.0
	1.16 (qd, *J* = 12.1, 4.0 Hz, 1H)	
11	1.77–1.71 (m, 1H)	35.7
12	2.90 (d, *J* = 9.5 Hz, 1H)	80.9
13		71.1
14	6.44 (dd, *J* = 10.1, 1.6 Hz, 1H)	131.3
15	5.62 (dd, *J* = 10.1, 5.3 Hz, 1H)	123.4
16	2.44 (dd, *J* = 5.3, 1.6 Hz, 1H)	50.7
17		85.0
18		205.2
19		132.0
20	6.57 (q, *J* = 7.3 Hz, 1H)	145.0
21	2.05 (d, *J* = 7.3 Hz, 3H)	16.5
22	1.22 (s, 3H)	19.0
23	1.04 (d, *J* = 6.2 Hz, 3H)	18.9
24	1.33 (s, 3H)	22.0
6-OH	4.03 (s, 1H)	
12-OH	1.85 (s, 1H)	
13-OH	1.70 (s, 1H)	

## Data Availability

The original contributions presented in this study are included in the article/Supplementary Material. Further inquiries can be directed to the corresponding author.

## Supplementary Materials

The following supporting information can be downloaded at: https://www.mdpi.com/article/10.3390/microorganisms14071430/s1, Figure S1: ^1^H NMR Spectrum of canakingmycin (CDCl_3_); Figure S2: ^13^C NMR Spectrum of canakingmycin (CDCl3); Figure S3: The 1H-1H COSY Spectrum of canakingmycin (CDCl_3_); Figure S4: The HSQC Spectrum of canakingmycin (CDCl_3_); Figure S5: The HMBC Spectrum of canakingmycin (CDCl_3_); Figure S6: The NOESY Spectrum of canakingmycin (CDCl_3_); Figure S7: The HRESIMS Spectrum of canakingmycin; Figure S8: The IR Spectrum of canakingmycin; Figure S9: The CD Spectrum of canakingmycin.
