# Supplementary material for: A Novel Skeleton Compound from Streptomyces canarius SN0246: Isolation, Purification, and Structural Elucidation
Source: Microorganisms. 2026 Jun 30;14(7):1430. doi: 10.3390/microorganisms14071430 (PMC13414243; doi:10.3390/microorganisms14071430)
Supplement: Supplementary file 1 [file microorganisms-14-01430-s001.zip › microorganisms-4383281-supplementary.pdf]

Content

Figure S1 ^{1}H NMR Spectrum of **canakingmycin** ($CDCl_3$)....1

Figure S2 ^{13}C NMR Spectrum of **canakingmycin** ($CDCl_3$) 1

Figure S3 The ^{1}H-^{1}H COSY Spectrum of **canakingmycin** ($CDCl_3$) 2

Figure S4 The HSQC Spectrum of **canakingmycin** ($CDCl_3$)....2

Figure S5 The HMBC Spectrum of **canakingmycin** ($CDCl_3$)....3

Figure S6 The NOESY Spectrum of **canakingmycin** ($CDCl_3$) 3

Figure S7 The HRESIMS Spectrum of **canakingmycin**....4

Figure S8 The IR Spectrum of **canakingmycin**....4

Figure S9 The CD Spectrum of **canakingmycin** 5

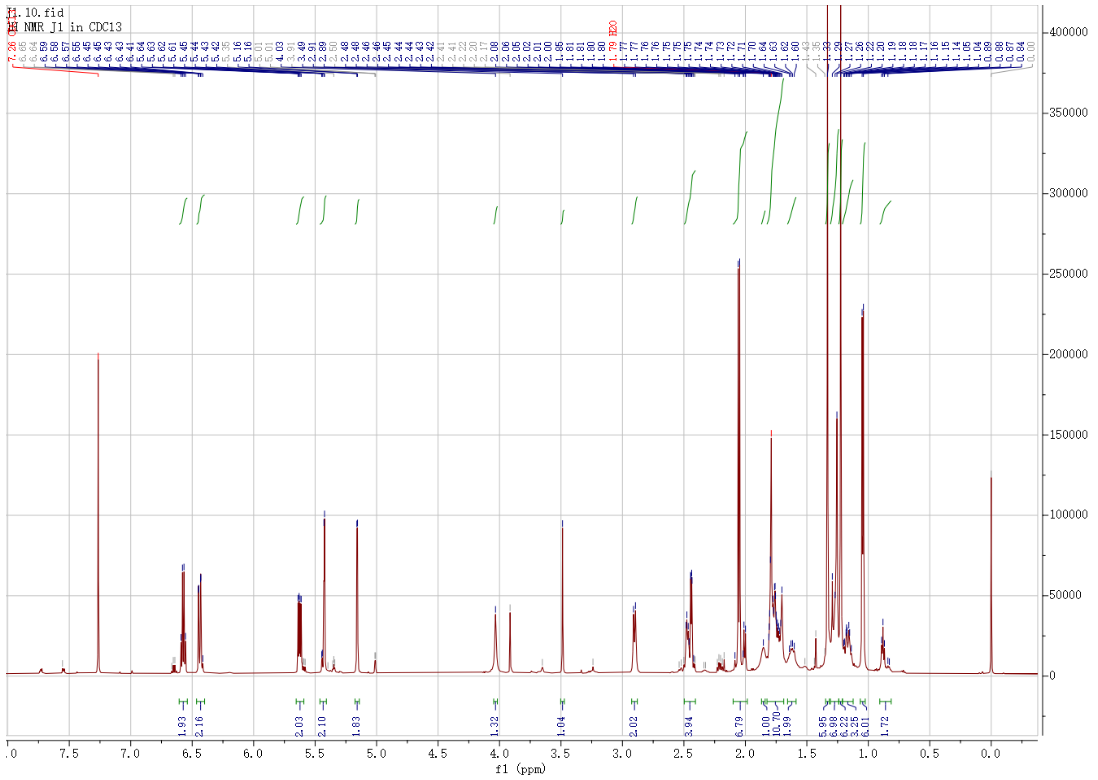

Figure S1 ^{1}H NMR Spectrum of **canakingmycin** ($CDCl_3$)

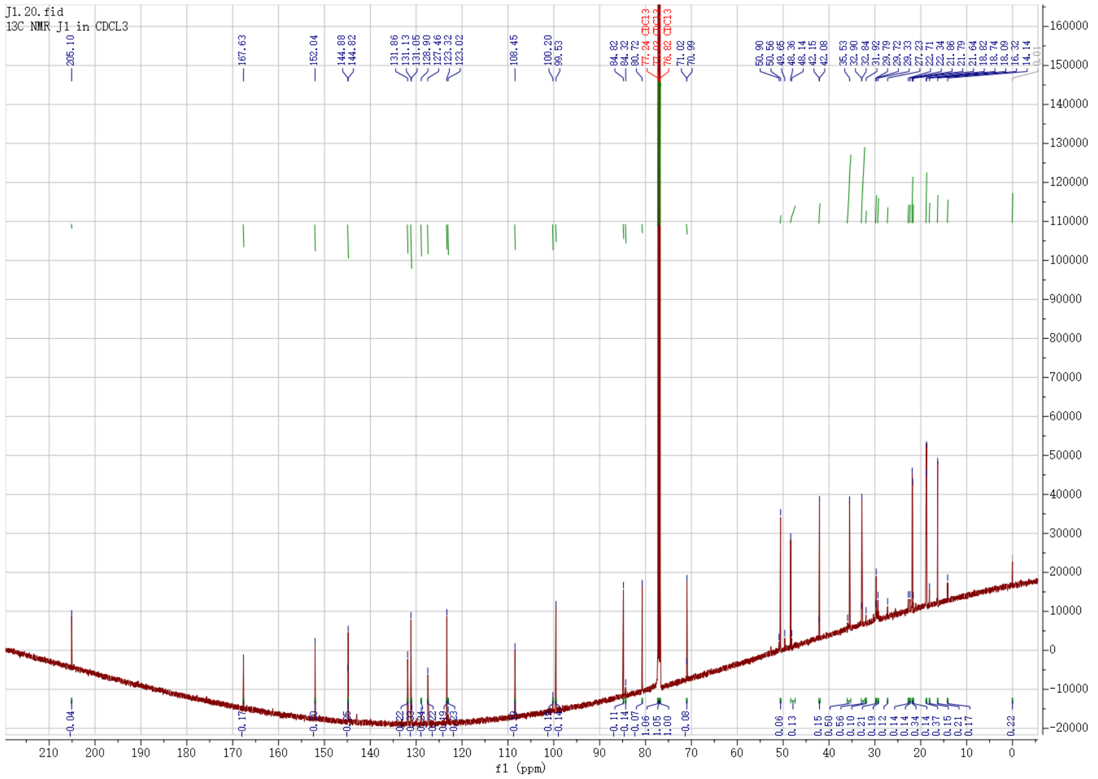

Figure S2 ^{13}C NMR Spectrum of **canakingmycin** ($CDCl_3$)

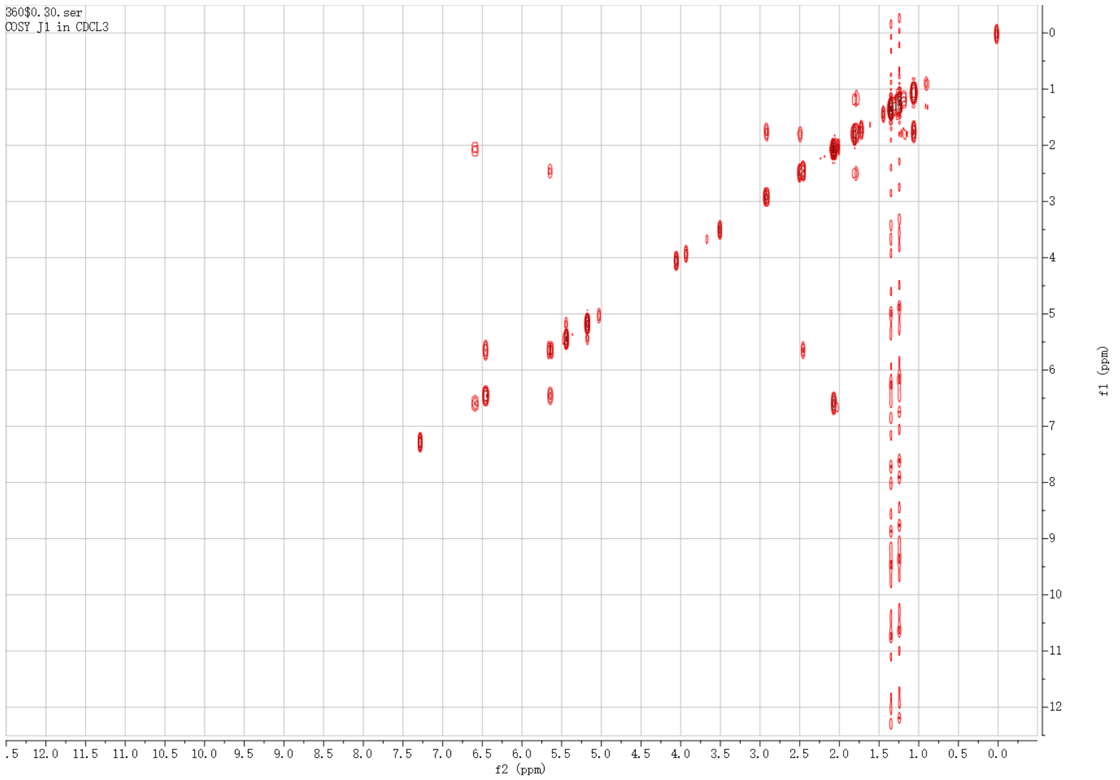

Figure S3 The ^{1}H-^{1}H COSY Spectrum of **canakingmycin** ($CDCl_3$)

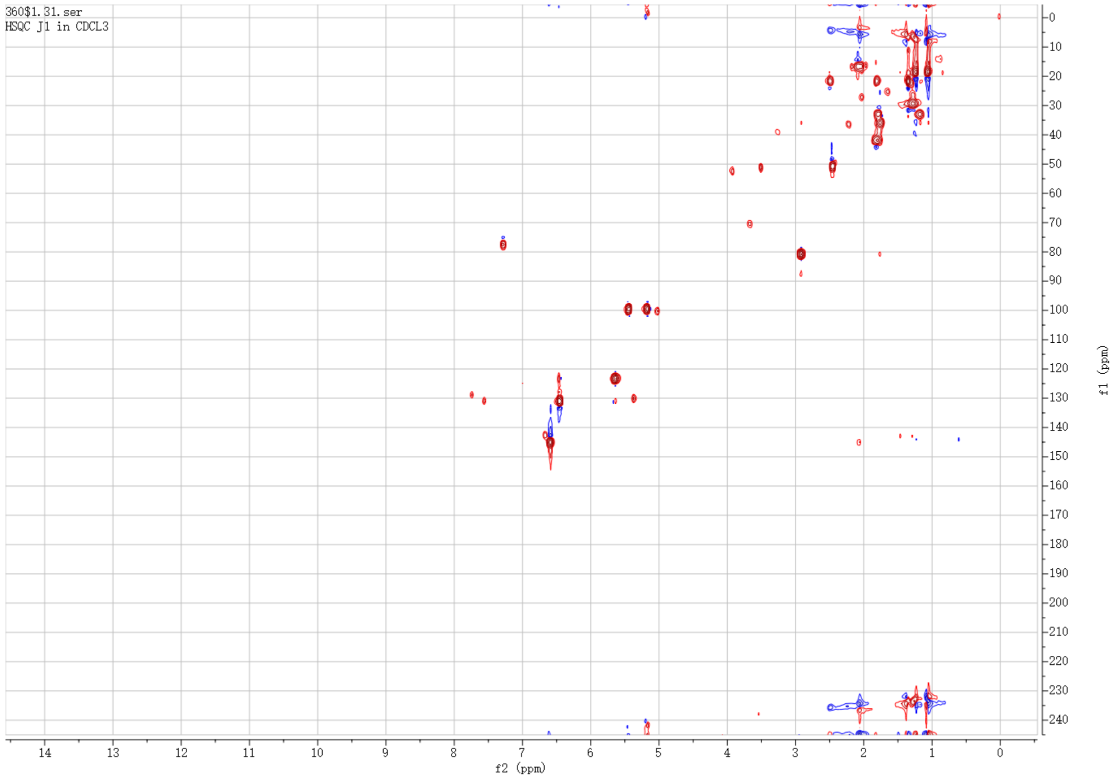

Figure S4 The HSQC Spectrum of **canakingmycin** ($CDCl_3$)

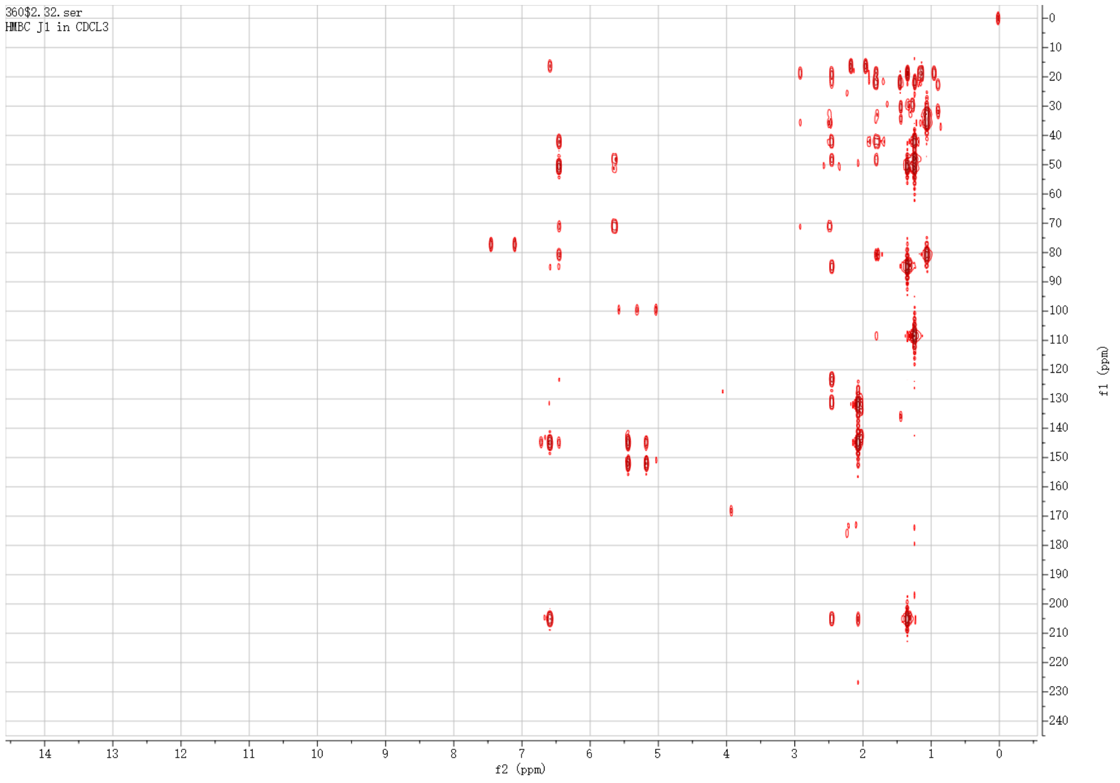

Figure S5 The HMBC Spectrum of **canakingmycin** ($CDCl_3$)

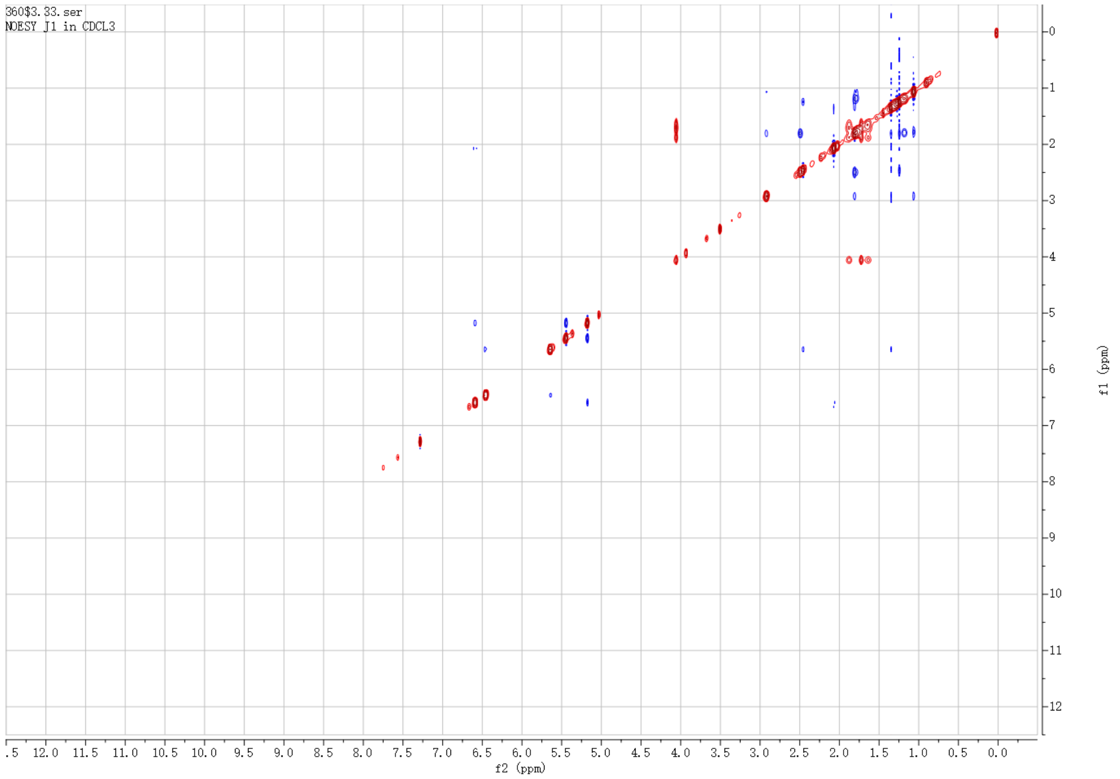

Figure S6 The NOESY Spectrum of **canakingmycin** ($CDCl_3$)

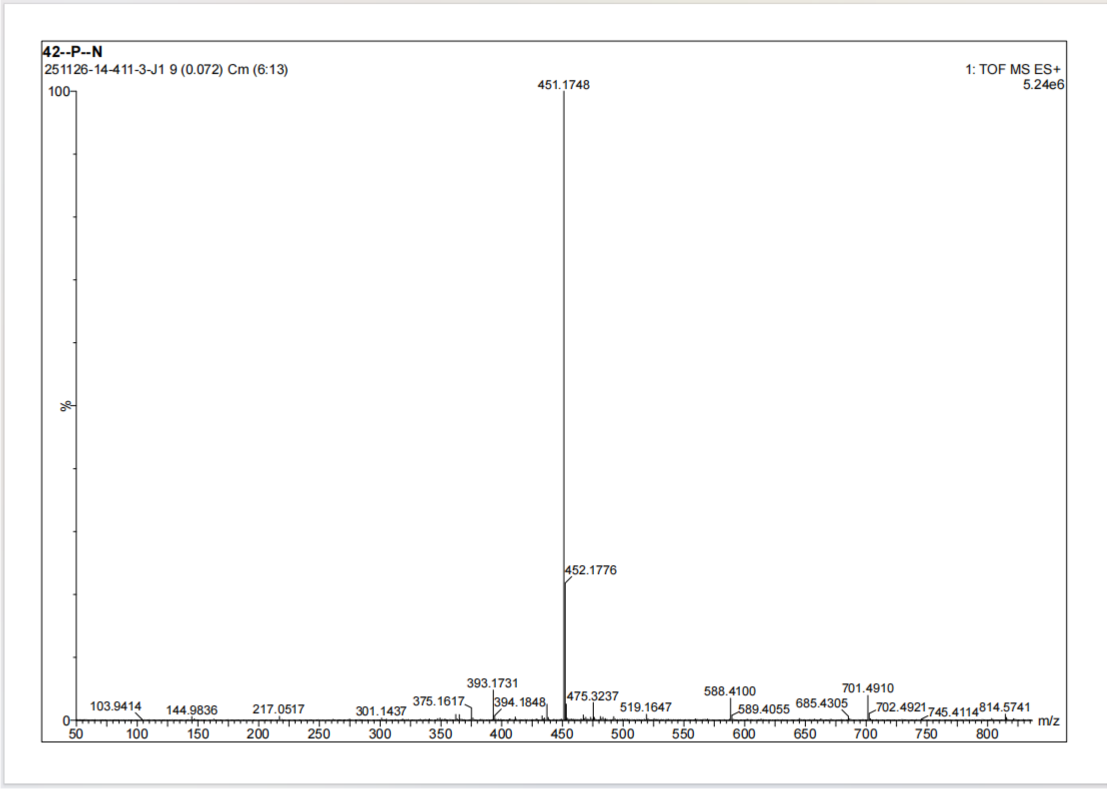

Figure S7 The HRESIMS Spectrum of **canakingmycin**

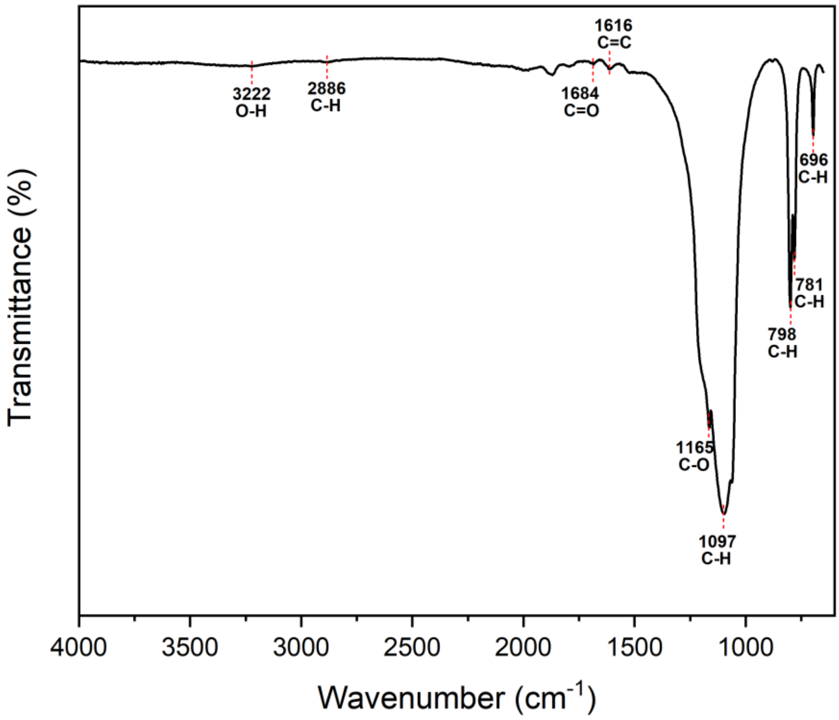

Figure S8 The IR Spectrum of **canakingmycin**

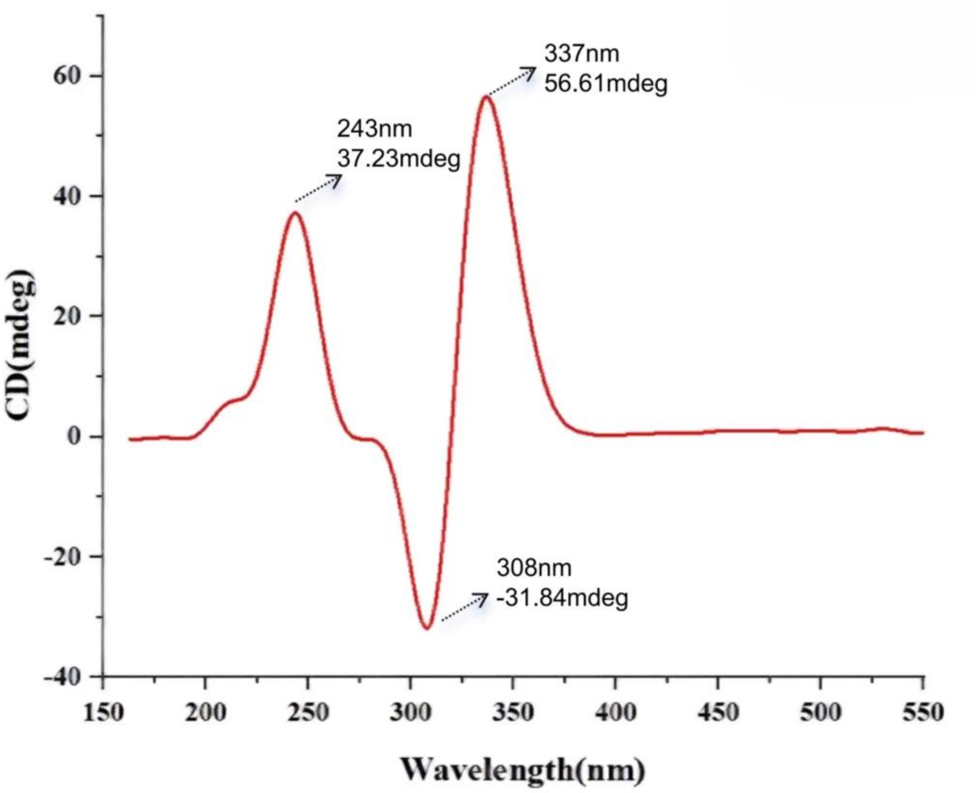

Figure S9 The CD Spectrum of **canakingmycin**
